# Dengue Virus Type 1 Infection in Traveler Returning from Tanzania to Japan, 2019

**DOI:** 10.3201/eid2509.190814

**Published:** 2019-09

**Authors:** Kazuma Okada, Ryo Morita, Kazutaka Egawa, Yuki Hirai, Atsushi Kaida, Michinori Shirano, Hideyuki Kubo, Tetsushi Goto, Seiji P. Yamamoto

**Affiliations:** Osaka Institute of Public Health, Osaka, Japan (K. Okada, K. Egawa, Y. Hirai, A. Kaida, H. Kubo, S.P. Yamamoto);; Osaka City General Hospital, Osaka (R. Morita, M. Shirano, T. Goto)

**Keywords:** dengue virus 1, Tanzania dengue, type 1, viruses, Tanzania, Japan

## Abstract

The largest outbreak of dengue fever in Tanzania is ongoing. Dengue virus type 1 was diagnosed in a traveler who returned from Tanzania to Japan. In phylogenetic analysis, the detected strain was close to the Singapore 2015 strain, providing a valuable clue for investigating the dengue outbreak in Tanzania.

Dengue fever is a febrile illness and a major public health problem caused by dengue virus (DENV), which infects almost 400 million persons worldwide every year (*1*). DENV has 4 serotypes (DENV-1–4), which are antigenically distinct. Although many countries in Africa are listed as being at a risk of DENV transmission, the molecular characterization of circulating DENV strains in these countries is poor.

In Tanzania, the number of patients with dengue fever increased sharply during April–May 2019, when >3,000 new suspected dengue cases were reported, including 2 deaths. Of these cases, 71.4% were confirmed by rapid diagnostic tests according to the World Health Organization; this total exceeded the previous worst dengue outbreak in 2014, which had 2,129 suspected and 1,018 confirmed cases (*2*). DENV-2 was reported as the causative agent of the 2014 outbreak in Tanzania (*3*,*4*), whereas DENV-3 exported from Tanzania was also documented (*5*). We describe the case of a traveler from Japan who was infected with DENV-1 in Tanzania amid the country’s largest dengue outbreak in 2019.

In early May 2019, a 32-year-old man who returned to Japan from Tanzania was admitted to Osaka City General Hospital in Osaka, Japan, after receiving a diagnosis of dengue fever at the Kansai International Airport quarantine station. He had been vaccinated against yellow fever before travel; additional test results were negative for chikungunya virus, Zika virus, and malaria. During his 10-day stay in Tanzania, the patient arrived at the airport in Dar es Salaam and stayed there during days 1–2 (specific locations in the Appendix Figure). On day 3, the patient flew to Kigoma, located in northwestern Tanzania; he reported being bitten by mosquitoes several times during the day there. On day 4, he visited Mahale Mountains National Park; the visit lasted for 3 days. On day 7, the patient returned to Kigoma and noticed fever and headache (disease onset). He traveled to Dar es Salaam on day 8 and stayed there for 2 days but was sick in bed at the hotel the entire time. He left Tanzania on day 10 to return to Japan and subsequently reported that he had been bitten by mosquitoes only in Kigoma.

We detected DENV-1 in the patient’s blood sample, collected 4 days after disease onset, using real-time reverse transcription PCR (RT-PCR) (*6*). We amplified a region of the envelope gene (1,485 nt) by RT-PCR using DENV-1–specific primers (*7*) and determined the sequence by direct sequencing (deposited in the DNA Data Bank of Japan as DV1/TZA/19RM-Osaka under accession no. LC485151). Through phylogenetic analysis based on a recent report (*8*), we classified the DV1/TZA/19RM-Osaka strain as genotype V and closely related to a 22125 strain in Singapore in 2015 with 98.6% nucleotide identity (Figure). The DV1/TZA/19RM-Osaka strain was distinct from other genotype V strains detected in Africa, suggesting that the DENV-1 strain in Tanzania might have been introduced from outside Africa.

**Figure:**
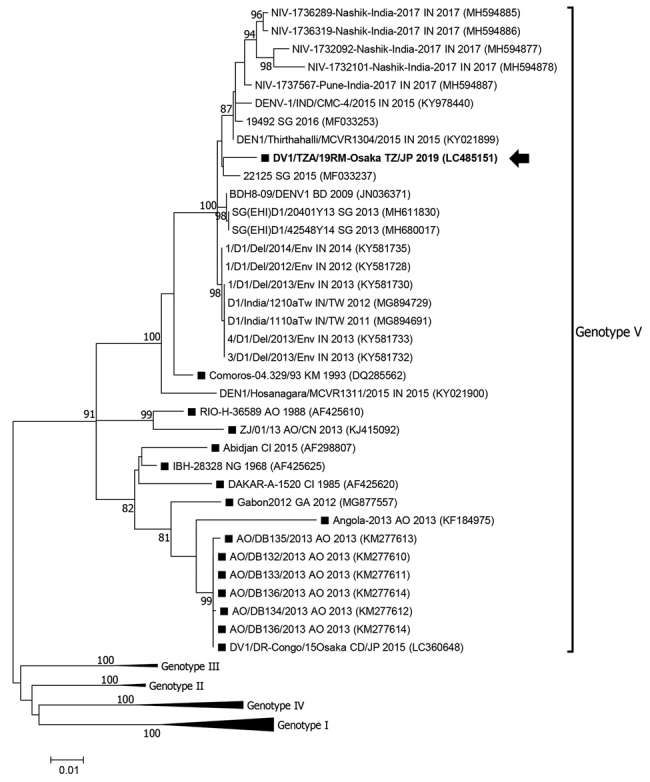
Maximum-likelihood phylogram of the envelope gene (1,485 nt) of dengue virus type 1 strains detected in Osaka, Japan, 2019 (arrow), Africa (black squares), and reference strains. Based on Bayesian information criteria, the Tamura-Nei plus gamma model was used to construct the phylogram. Numbers at the nodes indicate the bootstrap support values, which are expressed as a percentage of 1,000 replicates (values <80% are omitted). Each strain is identified by strain name, 2-letter country name abbreviation (country exported from/to, in the case of travelers), and detection year; accession numbers are shown in parentheses. Genotype I–IV branches are condensed for space. Scale bar indicates genetic distance (nucleotide substitutions per site). AO, Angola; BD, Bangladesh; CD, Democratic Republic of the Congo; CI, Côte d'Ivoire; GA, Gabon; IN, India; JP, Japan; KM, Comoros; NG, Nigeria; SG, Singapore; TW, Taiwan; TZ, Tanzania.

Dengue outbreaks occurred in 2010, 2012, 2013, and 2014 in Dar es Salaam, with the largest in 2014 (*3*). Recent studies have reported DENV-2 as the cause of the 2014 outbreak; the high similarity of the virus to DENV-2 strains from China, India, East Timor, and Singapore indicates that it might have been introduced by travelers from Asia (*4*). Another study indicated that DENV-3 was introduced or reintroduced in Tanzania from other countries in Africa or from the Middle East (*5*). In this study, we showed that the transmission of DENV-1, the genome of which was phylogenetically related to the strain derived in Singapore, has occurred in Tanzania.

Dar es Salaam has been the epicenter of the 2019 dengue outbreak and past outbreaks. From this patient’s mosquito bite history, we concluded that the patient was most likely infected with DENV-1 in Kigoma, but we cannot deny the possibility of unrecognized mosquito bites in Dar es Salaam. A recent study showed that 1% of dengue cases in the 2014 outbreak were reported from regions outside Dar es Salaam, including Kigoma, and model prediction suggested that Kigoma could have the possibility of occurrence of dengue concurrently with the dengue outbreak in Dar es Salaam (*9*). However, the disease was not detected on a large scale because of a lack of proper diagnosis (*9*), which suggests several unrecognized dengue cases in Kigoma even in the ongoing 2019 outbreak. Taken together, the DV1/TZA/19RM-Osaka strain may have originated from the outbreak strain in Dar es Salaam in 2019.

The phylogenetic relationships among DENV strains detected at different locations during the same epidemic in Tanzania are yet to be studied. Our data contribute to a better understanding of the epidemiology of DENV infections in Tanzania. Additional studies of dengue fever in Tanzania, not only in Dar es Salaam but also in other regions, would further clarify the epidemiology of this serious public health concern in this country.

Appendix Map showing the locations of travels in Tanzania for patient with dengue virus type 1 infection returning to Japan, 2019.
