# Supplementary material for: Dengue Virus Type 1 Infection in Traveler Returning from Tanzania to Japan, 2019
Source: Emerg Infect Dis. 2019 Sep;25(9):1782–4. doi: 10.3201/eid2509.190814 (PMC6711223; doi:10.3201/eid2509.190814)
Supplement: Appendix — Map showing the locations of travels in Tanzania for patient with dengue virus type 1 infection returning to Japan, 2019. [file 19-0814-Techapp-s1.pdf]

Article DOI: https://doi.org/10.3201/eid2509.190814

Dengue Virus Type 1 Infection in Traveler Returning from Tanzania to Japan, 2019

Appendix

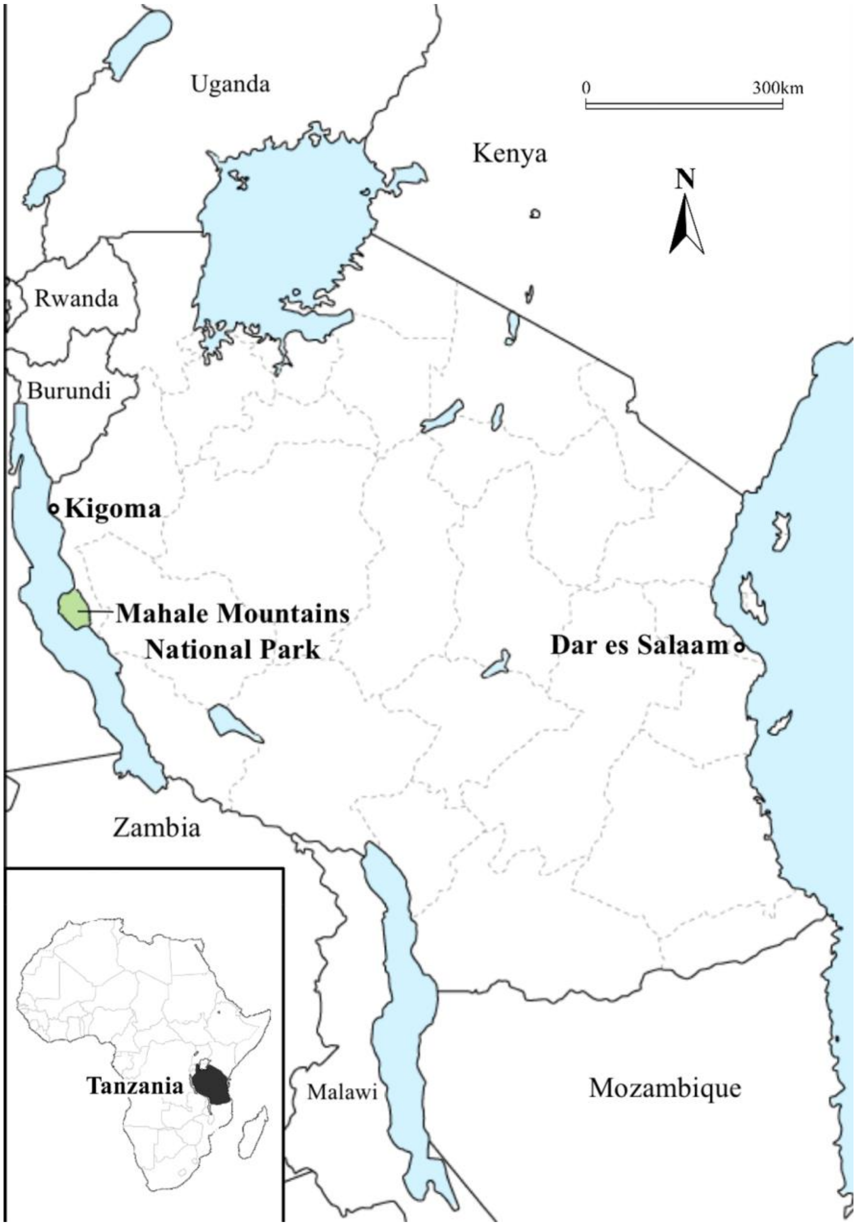

Appendix Figure. Map of Tanzania showing Dar es Salaam on the east coast and Kigoma and Mahale Mountains National Park in the northwest.
